# High Microbial Diversity Despite Extremely Low Biomass in a Deep Karst Aquifer

**DOI:** 10.3389/fmicb.2018.02823

**Published:** 2018-11-26

**Authors:** Olivia S. Hershey, Jens Kallmeyer, Andrew Wallace, Michael D. Barton, Hazel A. Barton

**Affiliations:** ^1^Department of Biology, University of Akron, Akron, OH, United States; ^2^GFZ German Research Centre for Geosciences, Potsdam, Germany; ^3^Department of Biological Sciences, Northern Kentucky University, Highland Heights, KY, United States; ^4^Joint Genome Institute, Walnut Creek, CA, United States; ^5^Department of Geosciences, University of Akron, Akron, OH, United States

**Keywords:** karst, cave, freshwater, aquifer, deep, ultra-oligotrophic, bacteria

## Abstract

Despite the importance of karst aquifers as a source of drinking water, little is known about the role of microorganisms in maintaining the quality of this water. One of the limitations in exploring the microbiology of these environments is access, which is usually limited to wells and surface springs. In this study, we compared the microbiology of the Madison karst aquifer sampled via the potentiometric lakes of Wind Cave with surface sampling wells and a spring. Our data indicated that only the Streeter Well (STR), which is drilled into the same hydrogeologic domain as the Wind Cave Lakes (WCL), allowed access to water with the same low biomass (1.56–9.25 × 10^3^ cells mL^-1^). Filtration of ∼300 L of water from both of these sites through a 0.2 μm filter allowed the collection of sufficient cells for DNA extraction, PCR amplification of 16S rRNA gene sequences, and identification through pyrosequencing. The results indicated that bacteria (with limited archaea and no detectable eukaryotic organisms) dominated both water samples; however, there were significant taxonomic differences in the bacterial populations of the samples. The STR sample was dominated by a single phylotype within the *Gammaproteobacteria* (Order *Acidithiobacillales*), which dramatically reduced the overall diversity and species richness of the population. In WCL, despite less organic carbon, the bacterial population was significantly more diverse, including significant contributions from the *Gammaproteobacteria, Firmicutes, Chloroflexi, Actinobacteria*, *Planctomycetes, Fusobacter*, and *Omnitrophica* phyla. Comparisons with similar oligotrophic environments suggest that karst aquifers have a greater species richness than comparable surface environs. These data also demonstrate that Wind Cave provides a unique opportunity to sample a deep, subterranean aquifer directly, and that the microbiology of such aquifers may be more complex than previously anticipated.

## Introduction

Karst is a term used to denote landscapes formed by water within soluble rock, often limestone (CaCO_3_). Aquifers that flow through karst landscapes often do so through solutionally enlarged conduits (caves), which provide multiple, direct connections between meteoric water, and groundwater systems (White, 1988; Ford and Williams, 2007). They also provide an important source of drinking water, with 25% of the world’s fresh water flowing through karst aquifers (Ford and Williams, 2007). Recent work has demonstrated that subsurface aquifers play an important, but overlooked, role in the global hydrologic cycle, contributing as much as four times the freshwater discharge into oceans as rivers and streams (Taniguchi et al., 2002; Kwon et al., 2014). Areas with significant karst landscapes, such as the Mediterranean Sea, can receive up to 75% of their freshwater input from karst springs rather than surface run-off (Garcia-Solsona et al., 2010). Aquifers also contribute to terrestrial nutrient input into the oceans (Garcia-Solsona et al., 2010; Weinstein et al., 2011; Kwon et al., 2014; McCormack et al., 2014). The importance of karst aquifers in geochemical cycles and as human water sources has led to an increase in research aimed at investigating indigenous microbial species in such groundwater (Farnleitner et al., 2005; Goldscheider et al., 2006; Griebler and Lueders, 2009; Pronk et al., 2009; Wilhartitz et al., 2009; Iker et al., 2010; Gray and Engel, 2013); however, the microbial ecology of these environments remains poorly understood, as the vast majority of karst groundwater research is concerned with point source contamination rather than native microbial community structure (Dojka et al., 1998; Boyer and Pasquarell, 1999; Cho and Kim, 2000; Ashbolt et al., 2001; North et al., 2004; John and Rose, 2005; Sinreich et al., 2014).

**FIGURE 1:**
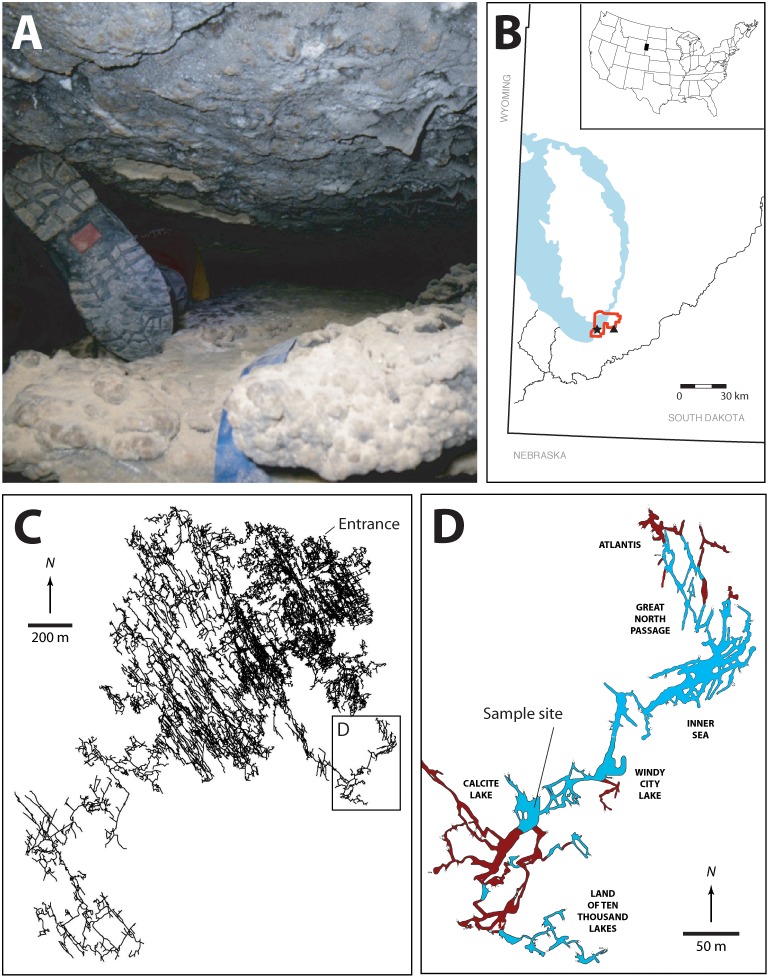
**(A)** Image illustrating some of the passages that must be traversed *en route* to the Wind Cave Lakes (it should be noted that this is not the smallest passage that researchers must navigate with equipment). **(B)** Location map of South Dakota, the Black Hills, and Wind Cave. The exposed Madison limestone, where some of the Madison aquifer water recharge occurs is indicated in blue, the location of Wind Cave National Park (red), Wind Cave (black star), and Streeter well (black triangle). **(C)** The survey line plot of the passages within Wind Cave, demonstrating the location of the lakes in relation to the natural entrance to the cave. **(D)** Location of the sample site within the lakes area. The lakes are indicated (in blue) along with dry cave passages (brown). The named areas of the cave are indicated. All arrows indicate true north. Cave data compiled by, and with permission of, Wind Cave National Park.

Wind Cave is found within the Mississippian age limestone of the Madison Formation along the eastern flank of the Black Hills South Dakota, United States (Figure 1). Originally designated a US National Park in 1903, Wind Cave is one of the longest (at 218 km) and oldest caves in the world, with initial cave forming processes (speleogenesis) occurring during the mid-Carboniferous period [∼350 million years ago (Mya); (Palmer and Palmer, 2000)]. The most recent speleogenetic processes began during the Eocene Epoch (∼50 Mya), when the Black Hills tilted toward the southeast, causing groundwater flow to accelerate passage enlargement and begin a close association between the aquifer and the cave (Bakalowicz et al., 1987). At a depth of -122 m, the cave intercepts the Madison aquifer, providing the only direct physical access (other than through drilled wells or discharge springs) to an enormous aquifer that underlies four United States and two Canadian provinces (Long et al., 2012).

At the point where Wind Cave intersects the aquifer a series of lakes are created (Figures 1C,D). The lack of an obvious discharge route out of the lakes and their relationship to the local potentiometric surface suggests that the surface of the lakes represents the local surface of the Madison aquifer (Back, 2011; Long et al., 2012). Measurements of stable isotopes in calcite precipitates near the lakes site suggest that they have existed in this region of Wind Cave for ∼1.14 (± 0.13) Myr, where they have remained isolated from diurnal or seasonal variation and under permanent aphotic conditions (Ford et al., 1993). Their geologic isolation also means that they remain separated from the surficial hydrologic cycle, with recharge water taking an estimated 25 years to reach the lakes (Back, 2011; Long and Valder, 2011). The lakes themselves sit in a region of the Madison aquifer containing groundwater flow paths in a complex aquifer pattern, with 39% recharge from the Precambrian rocks of the Black Hills, while 33 and 25% comes from ancient recharge basins (> 10,000 years) flanking the eastern and western slopes of the hills, respectively (Figure 1B; Long et al., 2012). This hydrology may explain the relative stability of the lake water chemistry; sampling over the past 40 years has demonstrated little variation in pH, electrical conductivity, temperature, nutrients (N and P), and dissolved O_2_ (Griebler and Lueders, 2009; Back, 2011). While presenting a variety of technical challenges for sample collection due to the significant distances from the surface, technical climbs and constricted passageways (Figure 1A), and depth underground, these lakes provide a rare and valuable window for directly accessing this region of the Madison aquifer.

Given the unique opportunity that Wind Cave provides to directly access an important aquifer, we examined the microbial diversity of the Wind Cave lakes (WCL) and compared it to microbial communities sampled from the aquifer by surrounding wells and springs. Our results suggest that the WCL contain a unique ultra-oligotrophic, deep subterranean lake ecosystem dominated by bacteria, with cell numbers well below those previously observed in similar freshwater environments, which cannot be directly sampled via regional wells and discharge springs.

## Materials and Methods

### Sample Sites and Sampling

Given their depth (-122 m) and distance (> 3 km) from the entrance, the WCL are subject to constant temperature (13.7°C air temperature; 13.8°C water temperature; Back, 2011), with no variation in relative humidity (99.9% measured using a RH300 Digital Psychrometer, Extech Instruments, Waltham, MA, United States). Park Well #2 (PW2), located within Wind Cave National Park (WCNP), was drilled as a source of drinking water, reaching a depth of -208 m and drawing water from the Madison aquifer. Streeter Well (STR) is a private well that is similarly drilled down into the Madison Formation, to a depth of -283 m (Long and Valder, 2011; Supplementary Figure S1). Before sampling, resident water was removed from the wells by flushing three well volumes (> 2,000 L) of water using hydrostatic pressure (Hose and Lategan, 2012; Smith et al., 2012). Beaver Creek Spring (BCS), is on private land to the south of WCNP was also sampled as part of this study (Long et al., 2012).

Given the remoteness of the cave sample site and the narrow size of cave passages traversed, all equipment had to be battery powered with its largest dimension no greater than the narrowest passage height (20 cm; Figure 1A). We therefore collected cells via filtration through a Nalgene disposable 0.2 μm filter unit using a SP200 variable speed peristaltic pump (Global Water Instrumentation, Gold River, CA, United States) with a pump rate of 1 L min^-1^ (Hershey et al., 2018). To filter at the wells, samples were collected using the same Nalgene filter set-up, with well water flowing into a 18 L, sterile, acid-washed bucket in which the filter unit was floated. To collect cells, the filter membranes were cut out of the filtration unit at the sample site using a sterile scalpel and stored in 70% alcohol for transport. Samples were stored at -80°C in the lab until processing.

### Chemistry

Inorganic water chemistry from each of the sample sites has been compiled from raw data provided by the National Park Service (United States) and (Back, 2011; Table 1). Water pH was measured in the field using an Accumet AP61 portable pH meter (Fisher Scientific, Pittsburg, PA, United States). Total organic carbon (TOC) analysis was carried out by WATERS laboratories (Western Kentucky University, Kentucky, United States) using high temperature combustion method (SM5310B) in a Shimadzu TOC analyzer TOC-V series. Due to the very low biomass observed in the lakes we decided to also measure (1,3)-(β)-D-glucan, a common polysaccharide of both Gram negative and positive bacteria, to assess total microbial biomass (Duenas et al., 2003). Quantification was carried out via a chromogenic method using the Glucatell reagent in a BioTek ELx808 microplate reader, with measurements averaged from samples collected over a two-year period.

**Table 1:** Water chemistry of the sample sites used within this study.

Site	pH	Temp	SO_4_ (mg/L)	As (μg/L)	NO_2_+NO_3_ (mg/L)	PO_4_^-^ (mg/L)	TOC (mg/L)	Glucan (pg/mL)
BCS	7.3	18.0	1,290	1.7	0.39	–	38.80	–
PW2	7.8	14.9	38.4	26.7	0.21	3.37	–	–
STR	7.6	12.8	11.4	8.5	0.51	2.44	34.58^a^	–
WCL	7.75	13.8	9.57	12.8	2.38	0.41	0.29^a^	*bdl*^b^


*^a^Average values of three separate samples. ^*b*^bdl – below detection limit.*

### Cell Counting

During four separate sampling campaigns (2009–2015), 10 mL samples of water were collected using aseptic technique and immediately preserved by the addition of 1 mL 20% paraformaldehyde, to a final concentration of 2%. Samples were kept on ice for transportation and were stored at 4°C until processing (generally < 1 week). For cell enumeration, samples were filtered onto a 25 mm diameter, 0.2 μm membrane filter (Anodisc or Cyclopore; Whatman, Piscataway, NJ, United States) and stained with SYBR Green I (unless stated otherwise, all chemicals were obtained from Sigma-Aldrich, St. Louis, MO, United States). Total cell counts were carried out using epifluorescence microscopy at 1000X, using either a Leica DMZ2500 (Leica, Wetzlar, Germany), or Olympus BX53 fluorescent microscope (Olympus America Inc., Center Valley, PA, United States). Blank counts were determined at the beginning and the end of each working day, and the average blank value (52 cells ml^-1^) was subtracted from each cell count. Between 50 and 200 fields of view were inspected in order to achieve a total count of at least 20 separate samples. To reduce cell aggregation and more accurately count cell numbers in the samples we used a number of techniques, including: (a) filtering the untreated sample straight onto the membrane, (b) immersing the sample vial into a sonication bath (Bandelin Sonorex, 10 min at 640 W) prior to filtration, (c) adding a detergent solution (100 mM disodium EDTA dihydrate, 100 mM sodium pyrophosphate decahydrate, 1 % vol/vol Tween 80) and methanol to the sample and vortexing it for 30 min to dissolve extracellular polymers; and (d) a combination of methods (b) and (c) (Kallmeyer et al., 2008). For detection of eukaryotic species, 10 L volumes of lake water were collected in triplicate and filtered onto a 25 mm diameter 0.2 μm or 8.0 μm filters, before staining with calcofluor white M2R and DAPI fluorescent stains (Sigma-Aldrich, St. Louis, MO, United States) following the manufacturers recommended protocol. Nucleated cells were counted for 50 fields of view on an epifluorescence microscope at 400X, as described. The absence of observable eukaryotic organisms was confirmed by scanning the membrane at 100X magnification.

For domain-specific cell counts, fluorescence *in situ* hybridization (FISH) was performed using the bacterial-specific primers EUB338, EUB338II, and EUB338III labeled with Cy3, and the archaeal-specific primers CREN499 and ARC915 labeled with Cy5. Details on the nucleotide sequence, specificities and formamide concentrations for the primers were used as described in probeBase (Loy et al., 2007). Briefly, 10 mL of paraformaldehyde-fixed water was filtered onto a 25 mm 0.2 μm Anodisc filter (GE Healthcare Bio-Sciences, Pittsburgh, PA) and stained with 50 ng/mL of each of the fluorescent probes as previously described (Daims et al., 1999). The filters were then enumerated on the Olympus BX53 fluorescent microscope. Samples were examined at a 1000X magnification with final counts being estimated from the average of 100 fields-of-view.

### Molecular Techniques

All DNA protocols were carried out in a laminar-flow hood using aerosol resistant pipette tips to reduce the likelihood of contamination, along with preparation controls (PCTL). These PCTL were created by subjecting all the sampling equipment to experimental processing in the absence of sample, including assembly and processing within the lab and at the field site. The PCTL were extracted in parallel with the samples throughout the DNA extraction, PCR amplified and pooled prior to pyrosequencing. Cells were dislodged from filters via vortexing in 10 mL of Buffer A [200 mM tris(hydroxymethyl)aminomethane pH 8.0, 50 mM ethylenediaminetetraacetic acid (EDTA), and 200 mM NaCl] with 0.3% wt/vol sodium dodecyl sulfate (SDS) as a surfactant, followed by centrifugation at 13,000 ×*g* (Barton et al., 2006). Genomic DNA was extracted from the pelleted cells using a low biomass bead beating protocol (Barton et al., 2006).

For pyrosequencing the DNA was PCR amplified using the universal primers 515F and 806R (Walters et al., 2011) in three separate reactions. Each sample was labeled with a unique barcode (Kozich et al., 2013) and the amplified DNA was sequenced using a Roche/454 pyrosequencer at University of Kentucky Advanced Genetic Technologies Center (UK-AGTC^1^). The resulting read data were analyzed using the QIIME software (Caporaso et al., 2010b) with nucleotide sequences from each site separated using the PCR-encoded barcode. Poor quality reads were filtered out using the QIIME 454 denoising pipeline (Reeder and Knight, 2010), and chimeric sequences were identified and removed using UCHIME (Edgar et al., 2011) *de novo* and reference-based detection. OTUs were identified using UCLUST (Edgar, 2010) and classified using the SILVA QIIME 16S database (SILVA 119; Quast et al., 2013). Sequences were aligned to the SILVA 119 core alignment using the QIIME PyNAST alignment function and used to generate an approximate maximum likelihood phylogenetic tree using the default FastTree parameters (Caporaso et al., 2010a; Price et al., 2010). Raw sequences for this study were uploaded to the NCBI sequence read archive (SRA) with the acquisition number SRP147561. Comparative 454-sequencing data sets from similar oligotrophic aquatic environments were obtained from the NCBI SRA, including accession numbers SRP010407, SRP058014, SRP021556, and ERP020663.

For alpha-diversity metrics, rarefied OTU tables were generated from sequence data in QIIME using a step size of 100 sequences and an even sampling depth of 3,900 sequences. Species richness for each sample was calculated using the Chao 1 non-parametric estimator over 10 randomized iterations of sampling, with the median values used for a best fit line generated in R (Chao, 1984; Venables and Ripley, 2002; Wickham, 2007, 2011). The species richness of each sample was calculated using average tables (including standard error) for Simpson’s Reciprocal Index generated in QIIME (Simpson, 1949; Caporaso et al., 2010b). Normalized OTU tables were generated for beta-diversity analyses utilizing weighted- and unweighted-Unifrac metrics for community comparisons and Principle Coordinate Analyses (PCoA) in R (Lozupone and Knight, 2005; McMurdie and Holmes, 2013).

## Results

There are a number of surface springs and wells near Wind Cave that provide access to water from the Madison aquifer (Long and Valder, 2011), of which two wells (PW2 and STR) and one spring (BCS), are found in in the same hydrogeologic domain as Wind Cave (as evidenced by the similarity in stable isotope values and chemistry to lakes found in the cave; Back, 2011). In order to determine how much water would need to be filtered to collect sufficient cells for DNA extraction, we carried out cell counts (Table 2). The lakes at WCL contained an average of 2.93 × 10^3^ cells mL^-1^, while STR and PW2 were slightly higher, at 6.30 × 10^3^ and 9.30 × 10^4^ cells mL^-1^, respectively (Table 2). These cell numbers are much lower than anticipated, as previous observations of other karst springs have cell counts in the range of 10^5^ - 10^6^ cells mL^-1^ (Farnleitner et al., 2005; Newton et al., 2011; Smith et al., 2012), lower than other measured bodies of water (Table 3). Indeed, these numbers are more comparable to much deeper (∼1,500 m) fracture fluids (McMahon and Parnell, 2014). The higher cell numbers seen in BCS (8.84 × 10^4^ cells mL^-1^; Table 2) may be due to its use as a water source for a large herd of cattle that periodically wander into the spring. Culture-based analyses on selective media indicated that this microbial population included members of the *Enterobacteriaceae* (data not shown), making it impossible to distinguish native from contaminant microorganisms, and no additional sampling was carried out at BCS.

**Table 2:** Comparative direct cell counts for the sites included in this study.

Sample	Treatment	Fields of view	Volume filtered (mL)	Cells/mL	Std Dev (%)
**All sample sites**					
CTL	None	200	5	5.2 × 10^1^	28.8
PW2	None	100	10	9.3 × 10^4^	4.2
BCS	None	100	10	8.8 × 10^4^	52.5
STR	None	100	10	6.3 × 10^3^	12.1
WCL	None	100	10	2.9 × 10^3^	8.2
**Calcite Lake**					
WCL	None	100	10	3.48 × 10^3^	10.8
WCL	None	100	10	1.47 × 10^3^	16.7
WCL	US	50	10	6.84 × 10^3^	24.3
WCL	US	100	10	1.88 × 10^3^	14.7
WCL	US	100	10	1.56 × 10^3^	16.2
WCL	Det. Mix	50	10	9.25 × 10^3^	20.9
WCL	Det. Mix	50	10	8.85 × 10^3^	21.3
WCL	Det. Mix + US	50	10	6.84 × 10^3^	24.3
WCL	Det. Mix + US	50	10	8.44 × 10^3^	21.8
**FISH**					
WCL	EUB338I/II/III	100	10	2.43 × 10^3^	18.3
WCL	CREN499/ARC915	100	10	3.48 × 10^1^	46.1
STR	CREN499/ARC915	100	10	*bdl^a^*	–
**Nucleated cells**					
WCL	None	100	10,000	0	0


*a = below detection limit.*

**Table 3:** Estimated cell counts in other aquatic environments.

Aquatic Environment	Depth (m below surface)	Typical bacterial abundance (cells/mL)	Reference
Open Ocean	Various depths	1.0 × 10^4^ – 1.0 × 10^7^	Whitman et al., 1998
Screened well, San Juan Basin, New Mexico	182–190	2 × 10^6^	Takai et al., 2003
CO_2_ Sink Reservoir, Ketzin, Germany	647	2–6 × 10^6^	Myrttinen et al., 2010
Eutrophic River Warnow	0	2.4 × 10^6^	Freese et al., 2006
North Atlantic	5–200	8.2 × 10^5^ – 2.4 × 10^6^	Rowe et al., 2012
West Pacific	5–200	2.9 × 10^5^–1.2 × 10^6^	Rowe et al., 2012
Crater Lake	0–200	2.0 × 10^5^ – 1 × 10^6^	Urbach et al., 2001
Sedimentary Rock Borehole, Hokkaido, Japan	0–482	4.61 × 10^4^–5.06 × 10^6^	Kato et al., 2009
Sargasso Sea	5–200	4.6–8.8 × 10^5^	Rowe et al., 2012
Bangomb site, Gabon, Africa	5–105	4.5 × 10^4^–5.8 × 10^5^	Pedersen et al., 1996
Artesian Well, Paris, France	800	1.0 × 10^4^–2.5 × 10^5^	Basso et al., 2005
Swiss Cave Pool	950	5.2 × 10^5^	Shabarova and Pernthaler, 2010
Limestone Karst Aquifer Spring	0	6.8 × 10^4^	Farnleitner et al., 2005
Dolomite Karst Aquifer Spring	0	1.5 × 10^4^	Farnleitner et al., 2005
Fault-bordered aquifer, Northern Japan	550	3 × 10^3^	Shimizu et al., 2006
WICA lakes	122	2.3 × 10^3^	This study
Lake Vostok	1500–2750	2.0 × 10^2^–1.0 × 10^3^	Karl et al., 1999
Dolomite, igneous rock, South African Mines	1700–3600	2.0 × 10^2^–3.4 × 10^3^	Borgonie et al., 2011


The comparatively high arsenic concentration in PW2 (Table 1), suggested that the water in this well was contaminated by a significant intrusion of water from the shallower Minnelusa aquifer (Supplementary Figure S1; Back, 2011), complicating the interpretation of the microbiology (PW2 was subsequently sealed and made inaccessible by WCNP due to high arsenic levels). We therefore focused our work on the samples obtained via the cave lake (WCL) and the accessible surface well (STR). To determine the contribution of archaea to these populations, we combined FISH with our cell counting methods. The results (Table 2) indicated that the Archaea were present in the WCL samples, albeit at a low rate (∼2%). We were unable to count archaea in the STR sample above background control values (Table 2). During our bacterial cell counts, no eukaryotes were visible in the WCL water samples. To confirm this, we counted cells in 10 L samples (Table 2). Although scant fungal spores (∼8–12 μm in diameter) were occasionally observed outside of the counting frame (data not shown), there were no identifiable eukaryotic/protozoan cells in WCL (Table 2). This lack of a eukaryotic population was supported by our inability to amplify any 18S rRNA gene sequences via PCR using the EK-1F/EK-1520 primer set (Lopez-Garcia et al., 2001; data not shown).

To determine whether the very low planktonic cell numbers in WCL represented a statistical outlier, samples were collected for counting from WCL over the course of six years and analyzed in two independent laboratories, using a variety of separation techniques to obtain more robust counting data. Our data (Table 2) indicated that cell numbers within WCL are relatively constant, with at an average of 2.48 × 10^3^ cells mL^-1^, although slightly higher numbers could be observed following efforts to disrupt biofilms (7.64 × 10^3^ cells mL^-1^; Table 2). The results from all methods across three separate sampling periods ranged from 1.56 to 9.25× 10^3^ cells ml^-1^, with untreated and sonicated samples being on the low end of the spectrum and detergent mix and detergent mix/ultrasonic treated samples being slightly higher (Table 2). As a secondary analysis to confirm these low cell numbers, we tested the water for total (1,3)-β-D-glucan, a common polysaccharide of both with Gram negative and positive bacteria. Our data demonstrates that the concentration of this polymer was below the limit of detection (< 31 pg mL^-1^). To convert this value to cell number, we compared it to (1,3)-β-D-glucan production in the model species, *Pediococcus sp.*, which generates an average level of 1.2 pg cell^-1^ (Duenas et al., 2003), indicating a detection limit of 31 ng equates to 2,500 cells mL^-1^. Thus, while quantitative (1,3)-β-D-glucan production by environmental species is unknown, our inability to detect this polymer supports the cell count data of low biomass (Table 2).

Cell counts from WCL and STR were used to determine the volume of water necessary to collect sufficient DNA for examining diversity in the samples: assuming approximately 3 fg DNA per cell and 2.93 × 10^3^ cells mL^-1^ in WCL, and a DNA extraction efficiency of 17% (Claassen et al., 2013), we determined that DNA analysis would require filtering a minimum of 200 L of water. We were able to filter ∼300 L of water from STR and WCL through a 0.2 μm filter, and obtained ∼5 ng of DNA from each site after extraction. In order to examine the microbial community profile at each site, we used 454-pyrosequencing of 16S rRNA PCR amplicons. The number of OTUs generated for analysis were distributed as follows: CL1: 5,566; CL2: 5,817; CL3: 4,340; SW1: 5,266; SW2: 5,706; SW3: 8,945; and PCTL: 3,983.

**FIGURE 2:**
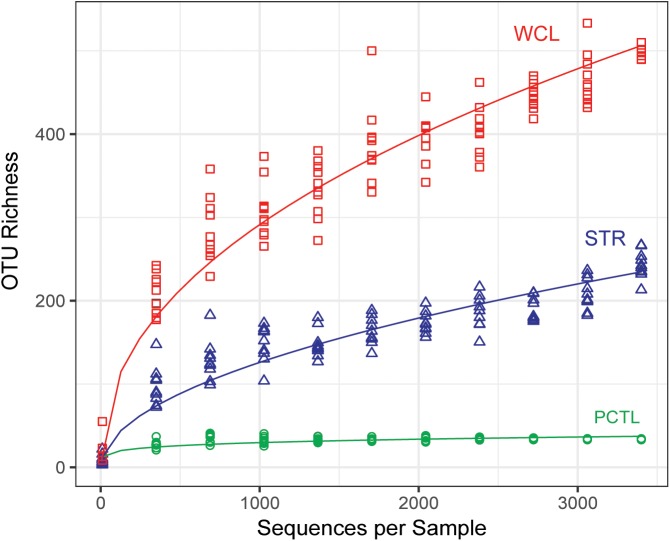
Chao 1 estimate of community species richness of WCL samples (red), STR samples (blue), and PCTL (green). Chao 1 points were generated using median abundance values of sample replicates, randomly subsampled (with replacement) at even sampling depths over 10 iterations. A best fit curve was generated using a robust linear model.

A rarefied analysis of the sequenced products (Chao, 1984; Figure 2) suggested that the WCL community demonstrates a significantly higher species richness and diversity than that found at STR, despite the 100-fold reduction in the amount of available organic carbon (0.29 mg L^-1^ versus 34.58 mg L^-1^). Indeed, the rarefaction curve for WCL suggests that the lakes contains hundreds of unique species, while STR phylotypes are more broadly represented within the analyzed data (as expected, there is limited diversity in the preparation control; Figure 2).

**FIGURE 3:**
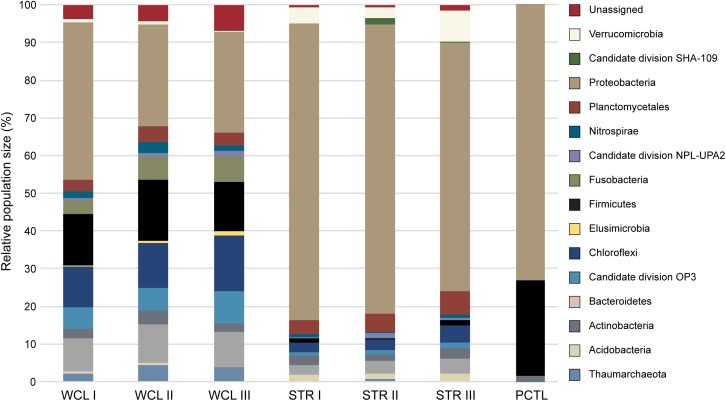
Phylum level 454-pyrosequencing data from the Wind Cave Lakes (WCL), Streeter Well (STR), and the processing control (PCTL). Three PCR replicates were performed for each sample. The relative contribution of each phyla is shown; phyla composing less than 1% of the microbial community were not included for clarity (a more detailed breakdown of all represented clades at the Order level is shown in Supplementary Table S1).

Analysis of the identified taxa revealed a broad phylum-level diversity within both the WCL and STR samples, with 14 and 11 represented phyla, respectively (Figure 3). The dominant phyla in WCL are similar to that observed in other cave biomes (Figure 3), with dominance by the *Proteobacteria* (averaged across three samples; 31%)*, Firmicutes* (14%), *Chloroflexi* (12%) and *Actinobacteria* (9%), along with significant contributions by members of the *Plantomycetes* (3.5%; Hershey and Barton, 2018). The WCL samples also contained a greater contribution by members of the *Fusobacter* (6%), *Omnitrophica* (7%), *Nitrospirae* (2%), and unclassified bacterial sequences (5%) compared to other cave samples (Figure 3). Sequencing demonstrated an average of 4% archaea within the WCL population, primarily consisting of the *Thaumarchaeota*, in support of the microscopic data (Table 2 and Figure 3).

Past hydrological analyses have suggested that STR well provides a convenient access point to sample the same aquifer as that found in the cave; however, the observed microbiology data does not support this. Rather, the STR sample demonstrated a reduction in total population of the *Actinobacteria* (averaging ∼3%), *Chloroflexi* (∼3%), *Firmicutes* (∼3%) and unclassified sequences (∼1%; Figure 3), with increases in the *Verrucomicrobia* (5%) and a dramatic increase by members of the *Gammaproteobacteria* (to as high as 73%). No significant archaea population (< 0.3%) was identified via sequencing in the STR sample (Figure 3). The preparation control sample (PCTL) contained far fewer sequencing products, with only three phyla present, including the *Proteobacteria, Firmicutes*, and *Actinobacteria*; this low diversity likely reflects the scant DNA amplified from pooled controls (Figure 3).

**FIGURE 4:**
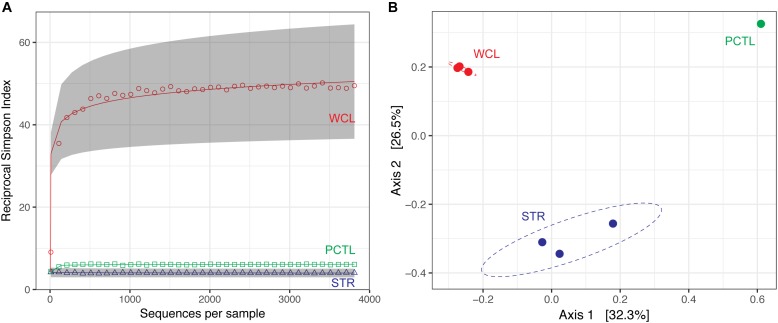
**(A)** Reciprocal Simpson rarefaction plot of WCL, STR, PCTL, and demonstrating community evenness. Data were randomly subsampled (with replacement) at even intervals to generate reciprocal Simpson curve (the 95% confidence intervals are indicated by gray shading). **(B)** Principle Coordinate Analysis (PCoA) of weighted Unifrac distances between WCL (red), STR (blue), and PCTL (green). Ellipses depict the 95% confidence interval of the sample cluster, with centroids determined by source mean values.

Despite the much lower available organic carbon in the WCL sample, the community appeared evenly distributed across several phyla, while STR is dominated by one phylotype (Figure 3 and Supplementary Table S1). In order to determine whether species richness was statistically different between samples, we used the reciprocal Simpson index to quantify the average proportional abundance of each taxa in the sample. The results (Figure 4A) suggest that there were more taxa represented in the WCL sample, with a higher proportional representation (richness) in the population (Interlandi and Kilham, 2001). This analysis confirms the observation that STR is dominated by one species. Indeed, even the minimal DNA amplified from the PCTL control appears more diverse than the community in STR (Figure 4A), suggesting that access to the organic carbon in STR may be selecting for this phylotype within the *Acidithiobacillales* (Figure 4A and Supplementary Table S1).

Population differences between the sample sites were further visualized using a weighted Unifrac principle coordinate analysis (PCoA; Figure 4B). The PCoA plot demonstrates that the WCL community is indeed significantly different from the STR (and PCTL) populations, with very little in-group difference between the WCL samples, compared with those for STR (Figure 4B). Thus, while WCL and STR had comparable, low cell numbers, the available organic carbon and/or geochemistry appears to have selected for quite distinct community compositions (Figures 3, 4).

**FIGURE 5:**
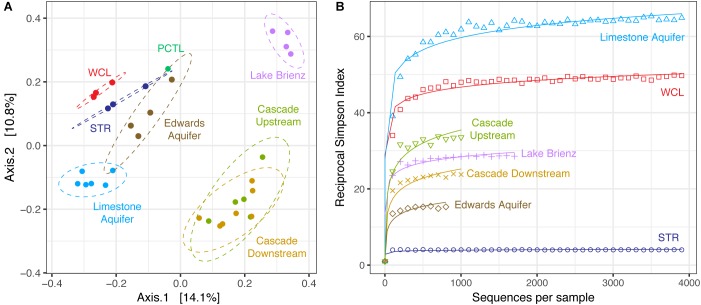
**(A)** Principle Coordinate Analysis plot of unweighted Unifrac distances between WCL, STR, PCTL, and similarly oligotrophic environments. Comparative data include the Edwards aquifer (SRP010407), pelagic microbial populations from oligotrophic Lake Brienz, Switzerland (SRP021556), a limestone aquifer (ERP020663), and stream water in a Kentucky cave (upstream and downstream; SRP058014). Ellipses depict the 95% confidence interval. **(B)** Comparative reciprocal Simpson rarefaction plot of WCL and STR, with the Edwards aquifer, Lake Brienz, a limestone aquifer, and the Kentucky cave stream, demonstrating community evenness.

To determine whether the WCL and STR populations shared similarity with other karst communities (despite being distinct from each other), we expanded our PCoA analysis to include comparable environments (Figure 5A). These sites were chosen based on oligotrophic conditions, sampling of pelagic microbial communities, and 454-pyrosequencing. These comparative samples included: the Edwards karst aquifer of Texas (Edwards aquifer; SRP010407; Engel and Randall, 2011); a shallow karst aquifer in Germany (Limestone aquifer; ERP020663; Herrmann et al., 2017); stream water in a Kentucky epigenic cave (Cascade Cave system; SRP058014; Brannen-Donnelly and Engel, 2015); and a surface lake open to photosynthetic input (the oligotrophic Lake Brienz; SRP021556; Köllner et al., 2013; Figure 5A). Analyses are therefore based on sequencing method (454-pyrosequencing), PCR primers and average number of amplicons.

Three different distance methods (normalized-weighted Unifrac, unweighted Unifrac, and the non-phylogenetic Bray-Curtis metric) were used to generate distance matrices for PCoA to visualize differences between the samples in this study and the data from the comparable environments. All metrics used generated similar grouping patterns in a principle coordinate analysis; due to the varying samples sizes used among studies we used the unweighted Unifrac metric (which is qualitative, rather than quantitative; Figure 5A). This PCoA demonstrates that the karst aquifer/spring samples exhibit similarity in the taxa identified, grouping together and remaining distinct from both the epigenic cave stream and surface lake samples (Figure 5A); however, even with this grouping the WCL and STR populations remain distinct from other samples, although differences in the primer sets used (V1/V3 versus V3/V4) may influence the overall result. In order to determine whether there were similarities in species richness between the karst aquifers, we again we used a comparable Reciprocal Simpson index, with a best fit projection for samples with fewer available sequences (Figure 5B). The results demonstrated that the site that shares the closest hydrogeological similarity to WCL, the German limestone aquifer, also demonstrated high species richness. In all cases, STR had the lowest overall diversity, despite being structurally similar to WCL in terms of geologic isolation and aphotic conditions (Figure 5B).

## Discussion

Wind Cave is one of the most geologically complex caves in the world, the structure of which provides the rare opportunity to travel into the subsurface and directly sample the microbiology of the Madison aquifer (Figure 1; Palmer and Palmer, 2000). Given the physical and technical challenges of examining the aquifer via the cave (Figure 1A), we wanted to examine how the microbiology sampled through the cave compared to the more easily accessible surface wells (PW2 and STR) and spring (BCS). Both PW2 and BCS contained a higher cell number than either WCL or STR (Table 2); however, high levels of As and SO_4_^2-^ in PW2 and BCS indicated that these sites were heavily influenced by the shallower Minnelusa aquifer (Table 1 and Supplementary Table S1).

Due to the chemistry at PW2 and BCS, STR has traditionally been used by hydrologists to sample the Madison aquifer (Back, 2011). Despite purging more than three volumes (2,000 liters) of water from STR (as recommended in past protocols; Hose and Lategan, 2012; Korbel et al., 2017), our data demonstrated differences between the STR and WCL samples (Figures 3, 4). These included a higher level of TOC (a variable not examined in previous hydrologic studies) and SO_4_^2-^ at STR compared to WCL. We believe that these higher values may be due to minimal casing of the STR well (Ohms, M., personal communication, 2018), which would allow water from the shallower Minnelusa Formation to be drawn by the well (Back, 2011; Long and Valder, 2011). Thus, while the main volume of water from STR may be from the same hydrogeologic domain as Wind Cave, it is mixing with the higher organic content, SO_4_^2-^, and microbiology of the Minnelusa (Supplementary Figure S1; Atlas et al., 1991; Naus et al., 2001).

There appears to be microbiological evidence for this water mixing within the STR well. While total cell numbers were comparable between the two sites (6.3 × 10^3^ cells mL^-1^ at STR versus 1.56–9.25 × 10^3^ cells mL^-1^ at WCL; Table 2), taxonomic comparisons demonstrated that the microbial populations were significantly different (Figure 3). While both the WCL and STR samples were both dominated by members of the *Gammaproteobacteria*, at the order level there were important differences (Supplementary Table S1). Within WCL, the *Gammaproteobacteria* (18% of identified OTUs) were dominated by members of the *Pseudomonadales, Xanthomonadales*, and *Chromatiales.* In STR, the much higher *Gammaproteobacterial* population (∼73%), comprised a single OTU within the *Acidithiobacillales* (Figure 3 and Supplementary Table S1). This OTU had the greatest sequence identity to an uncultured *Acidithiobacillales* clone KCM-B-112, which has been found in environments with high hydrocarbon content (Garrity et al., 2005; Marti et al., 2017). While this suggests that hydrocarbon breakdown may drive community energetics within STR, the closest cultured species, *Thioalkalivibrio sulfidiphilus*, is a chemolithotrophic, sulfur-oxidizer, suggesting that S-cycling may also be important (Muyzer et al., 2011). Nonetheless, *T. sulfidiphilus* only has 89% 16S sequence identity to KCM-B-112, and the low SO_4_^2-^-levels in STR make such metabolic inference difficult without additional biogeochemical support. The presence of similar members of the *Acidithiobacillales* at low abundance (< 0.2%) in the WCL samples, suggests that STR may be seeded by microorganisms from the aquifer, but are selected for by the geochemistry of the well environment or grow within the Minnelusa Formation (Figure 3 and Supplementary Table S1). In contrast, WCL is completely contained within the Madison limestone formation and not exposed to the organic content of the Minnelusa Formation (Supplementary Figure S1). Indeed, the TOC in WCL is much lower (0.29 mg L^-1^) compared to STR at 34.59 mg L^-1^, although the SO_4_^2-^ is comparable (Table 1). Our work supports the findings of Korbel et al. (2017), who determined that despite purging of stagnant water from wells and boreholes, microbial community analyses can still be influenced by variances in well casings, geologic strata through which wells pass, and even the purging strategies used (Hose and Lategan, 2012; Korbel et al., 2017). Thus, while STR may be a more easily accessible sample site for access to the water chemistry of the Madison aquifer, accurate microbiological investigations of this important water source require sampling via the cave. Consequently, the samples collected through the cave provide the most accurate window into the microbiology of the Madison aquifer.

At the phylum level, in addition to *Gammaproteobacteria*, the WCL community contained members of the *Actinobacteria* and *Firmicutes*, and significant contributions from the *Planctomycetales, Nitrospirae, Fusobacteria, Chloroflexi* and *Omnitrophica* (Figure 3). The *Actinobacteria* and *Firmicutes* are dominated by members of the *Actinomycetales, Bacillaceae, Pseudomonadales*, associated with carbon turnover in soils (Barton, 2015). The high levels of NO_2_^-^+NO_3_^-^ present in the lakes may support the growth of autotrophic nitrite oxidizing *Nitrospirae* (Lücker et al., 2010; Daims et al., 2015). It is unclear as to a potential source of this NO_3_^-^ in WCL; however, the nearby South Dakota Badlands are comprised of caliche, which are nitrate-rich paleosols deposited when conditions were more arid in this semi-arid region. Given the close association of the Badlands with a local recharge zone or the potential for deep burial, it may be that the waters pass through such deposits *en route* to WCL. Members of the *Nitrospirae* are primarily associated with nitrite oxidation, with some members capable of complete nitrification of ammonia to nitrate (Daims et al., 2015). The *Nitrospirae* have also been shown to be slow growing and metabolically flexible, with the ability to grow mixotrophically under microaerophilic or anaerobic conditions (Fujitani et al., 2014; Koch et al., 2015). Together with the presence of ∼2% ammonia-oxidizing *Thaumarchaeota*, this may indicate an active nitrogen cycle driving autotrophic growth within the lakes (Figure 3).

With limited or no cultured representatives, the metabolic activity of the *Planctomycetales, Chloroflexi* and *Omnitrophica* make their activity in the environment difficult to predict. Nonetheless, these phyla are often highly represented in oligotrophic environments, particularly caves, where they appear to grow using oxidized-carbon compounds under extreme nutrient-limited conditions (Fuerst and Sagulenko, 2011; Barton, 2015). Members of the *Omnitrophica* are widely distributed in subsurface environments (Rinke et al., 2013), but their potential metabolic role is poorly understood (Glöckner et al., 2010; Shabarova and Pernthaler, 2010; Momper et al., 2017). Recent work suggests that members of the *Omnitrophica, Nitrospirae* and *Planctomycetales*, are involved in iron-oxidation, and may play important roles in the geochemical cycling of iron and sulfur in the environment (Lefèvre, 2015; Lin et al., 2017; Momper et al., 2017). These data, along with the observation of other iron-cycling genera, such as *Geothrix*, suggest that iron (which is abundant within Wind Cave) may be an important driver of autotrophic growth (Kolinko et al., 2011; Lefèvre et al., 2013). These data suggest that autotrophic growth within WCL may be driven by iron, nitrogen and carbon cycling, similar to that observed in similar German karst aquifer and the nearby deep Sandford Underground Research Facility (Herrmann et al., 2017; Momper et al., 2017).

At the phylum level, the taxa observed in WCL appeared to be similar to that seen in other karst environments (aquifers and springs) and remain distinct from communities influenced by surface nutrient sources, such as Lake Brienz (Figure 5A). The WCL samples cluster together with the other karst aquifers, but remain distinct from the epigenic cave stream (created where surface waters flows into the subsurface; Figure 5A). The STR sample intersects within the 95% confidence interval of the Edwards aquifer data, which intersects with the German limestone aquifer. This is likely due to similar sampling strategies, with the German limestone aquifer and Edwards aquifer using well access and similar primer sets; however, humanly accessible caves do not intersect these aquifers at depth, preventing in-cave sampling (Engel and Randall, 2011; Herrmann et al., 2017), while the limited access to WCL has prevented re-sampling with the same primer sets to confirm this grouping. In the PCoA plot, the PCTL sample was close to the confidence intervals of two of the sampled sites (Figure 5A). Although this clustering may be due to resolution (when compared to WCL and STR alone, the PCTL sample was quite distinct), it suggests that control sample data from low biomass samples remain an important dataset to demonstrate the absence of sample/sequencing contamination (Barton et al., 2006). The PCTL itself was dominated by members of the *Legionella*, which generally live inside amoebae in the natural environment (Harf and Monteil, 1988). We were unable to detect eukaryotic microorganisms within the lake via PCR amplification or direct microscopic observation, and the dominance of *Legionella* in the preparation control may reflect contamination of purchased buffers (DNase- and RNase-free laboratory water controls did not contain such contamination). Such contamination has been shown to be present in commercial laboratory reagents, further emphasizing the importance of PCTL when working with low biomass samples (Shen et al., 2006).

One of the most notable observations from this study was that the WCL samples, under the lowest nutrient conditions, had amongst the highest observed diversity (Figure 5B). This has been observed before: described by Hutchinson as the “*paradox of the plankton*” (Hutchinson, 1961). Microbial ecologists have attempted to describe the differences that account for this phenomenon, arguing that environmental heterogeneity, mixing and even predation prevent the establishment of true competitive exclusion, allowing for high species richness even in the most starved environments (Czaran et al., 2002; Kerr et al., 2002; Torsvik et al., 2002; Cadier et al., 2017). Such obvious mechanisms of heterogeneity are absent at the lakes within Wind Cave: the cave has stable environmental conditions, with no diurnal, seasonal or annual variation; the cave entrance is miles from the sample site, and there is no airflow sufficient to facilitate lake water mixing; and no potential eukaryotic predators/grazers were detected (Table 2). An alternate explanation is that of Interlandi and Kilham (2001), who examined planktonic diatom diversity in oligotrophic lake systems. Their work demonstrated a correlation between the number of limited resources, biomass and diversity (measured using the Simpson’s index; Interlandi and Kilham, 2001). These investigators argued that resource competition itself was driving high diversity: despite the bulk water chemistry being homogenous, at the scale of individual microbial cells, there is sufficient resource variation to promote competition between species (even in low biomass environments; Interlandi and Kilham, 2001). The absence of light, low biomass, ultra-oligotrophic conditions, and limiting P may therefore contribute to the high diversity observed within both WCL and the German limestone aquifer (Figure 5B; Herrmann et al., 2017).

This work represents the first study of the microbiology of a deep, geologically isolated aquifer sampled directly via access through a cave. Our data suggest that Wind Cave provides a unique access point to study the microbial community of an aquifer without the confounding variables introduced via well or spring sampling (Yanagawa et al., 2013). The data suggest very different community energetics than would have been suggested through sampling a well and indicate that the microbial communities have a much higher potential to influence the quality of drinking water than would have been determined from well-based analyses (for example, denitrification; Herrmann et al., 2017). Nonetheless, the growing population and development of the Black Hills region means that demand on the Madison aquifer has increased. Recent permits have been approved to increase the amount of water drawn from the STR to ∼91,000,000 L year^-1^, with additional wells in the Black Hills and Fall Rivers water districts predicted to draw an additional ∼1,420,000,000 L year^-1^. If these wells draw water faster than the local recharge rates, this could result in a dramatic drop in the potentiometric surface of the Madison aquifer (Greene, 1993). If the aquifer were to drop below the current level of the cave, access via Wind Cave (and the unique ability to sample the microbiology independent of well access) will be gone. It is therefore critical to carry out as many microbiological analyses of this important aquifer before the site is lost.

## Author Contributions

OH, HB, and JK wrote the manuscript. Sample collection, processing and analysis were carried out by OH, JK, AW, MB, and HB, with bioinformatics analysis by OH with MB.

## Conflict of Interest Statement

The authors declare that the research was conducted in the absence of any commercial or financial relationships that could be construed as a potential conflict of interest.

## Footnotes

1 https://ukhealthcare.uky.edu/genomics-core-laboratory
