# Supplementary material for: High Microbial Diversity Despite Extremely Low Biomass in a Deep Karst Aquifer
Source: Front Microbiol. 2018 Nov 26;9:2823. doi: 10.3389/fmicb.2018.02823 (PMC6275181; doi:10.3389/fmicb.2018.02823)
Supplement: Supplementary file 1 [file Data_Sheet_1.PDF]

High microbial diversity despite extremely low biomass in a deep karst aquifer

Olivia S. Hershey[1], Jens Kallmeyer[2], Andrew Wallace[3], Michael D. Barton[4] and Hazel A. Barton[1,5*]

Supplemental Information

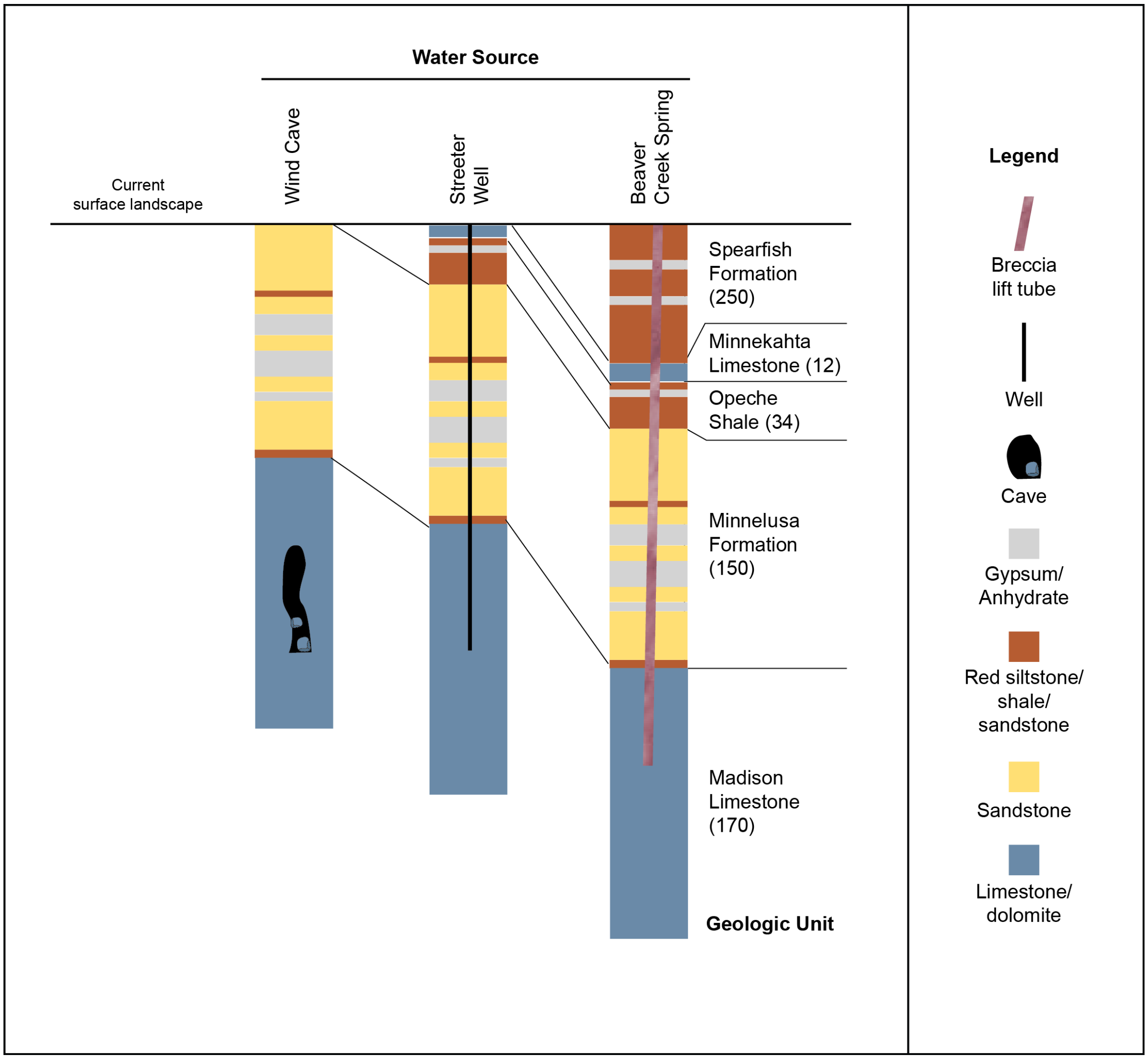

Supplemental Figure S1. Geologic profile of sample sites. Stratigraphic profile demonstrating the overlying rocks that confine the Madison aquifer. WICA is located within the Madison Limestone Formation. STR is completed to the Madison aquifer, and passes through the Minnelusa Formation (sandstone and shale), as well as the Opeche Shale. STR is a partially cased well. Beaver Creek Spring (west of WICA and STR) is located in the Spearfish Formation, with water originating from the Madison aquifer. Adapted from Epstein 2001, and Long and Vader, 2011.
