# Supplementary material for: High Microbial Diversity Despite Extremely Low Biomass in a Deep Karst Aquifer
Source: Front Microbiol. 2018 Nov 26;9:2823. doi: 10.3389/fmicb.2018.02823 (PMC6275181; doi:10.3389/fmicb.2018.02823)
Supplement: Supplementary file 2 [file Data_Sheet_2.PDF]

Supplemental Table S2: Order-Level Diversity (%) Pyrosequencing Data*

				Sample Site						
**Domain**	**Phylum**	**Class**	**Order**	**WCL I**	**WCL II**	**WCL III**	**PCTL**	**STR I**	**STR II**	**STR III**
Archaea										
	Euryarchaeota									
		Halobacteria								
			Halobacteriales	0.14	0.35	0.44	0.00	0.02	0.00	0.01
		Thermoplasmata								
			Thermoplasmatales	0.29	0.41	0.41	0.00	0.00	0.14	0.03
	Thamarchaeota									
			Unclassified	0.29	0.21	0.32	0.00	0.00	0.00	0.00
		Marine Group I								
			Unclassified	0.83	2.48	0.90	0.00	0.17	0.61	0.00
		Soil Crenarchaeotic Group SCG								
			Unclassified	0.13	0.00	0.05	0.00	0.04	0.00	0.10
		South African Gold Mine Gp 1 (SAGMCG-1)								
			Unclassified	0.43	1.10	1.68	0.00	0.02	0.00	0.15
		Unclassified		0.31	0.48	0.65	0.00	0.00	0.00	0.00
Bacteria										
	Acidobacteria									
		Acidobacteria								
			Acidobacteriales	0.00	0.02	0.00	0.00	0.19	0.07	0.11
			Subgroup 17	0.05	0.22	0.00	0.00	0.17	0.00	0.19
			Subgroup 25	0.00	0.00	0.00	0.00	0.17	0.00	0.02
			Subgroup 3	0.38	0.07	0.18	0.00	0.10	0.11	0.07
			Subgroup 4	0.05	0.12	0.00	0.00	0.76	1.02	0.84
			Subgroup 6	0.02	0.03	0.00	0.00	0.13	0.26	0.45
	Actinobacteria									0.00
		Acidimicrobiia								
			Acidimicrobiales	0.09	0.45	0.16	0.00	0.25	0.00	0.35
		Actinobacteria								
			Actinomycetales	2.16	3.00	2.79	0.00	0.00	0.00	0.00
			Corynebacteriales	0.67	0.26	0.07	0.08	0.21	1.16	0.11
			Frankiales	0.00	0.00	0.00	0.00	0.06	0.33	0.25
			Glycomycetales	0.02	0.03	0.39	0.00	0.00	0.00	0.00
			Kineosporiales	0.00	0.00	0.00	0.00	0.00	0.05	0.13
			Micrococcales	3.50	3.78	3.02	0.13	0.21	0.00	0.66
			Micromonosporales	0.00	0.03	0.00	1.78	0.11	0.67	0.16
			Propionibacteriales	1.12	0.91	1.31	0.40	0.19	0.00	0.18
			Pseudonocardiales	0.45	0.60	0.71	0.00	0.55	0.04	0.85
			Streptomycetales	0.09	0.00	0.18	0.08	0.04	0.00	0.12
			Streptosporangiales	0.27	0.00	0.09	0.00	0.02	0.00	0.01

	MB-A2-108								
		Unclassified	0.07	0.12	0.00	0.00	0.19	0.58	0.27
	Nitriliruptoria								
		Euzebyales	0.11	0.05	0.14	0.00	0.06	0.00	0.00
		Nitriliruptorales	0.11	0.45	0.37	0.00	0.00	0.00	0.00
	TakashiAC-B11								
		Unclassified	0.00	0.00	0.00	0.00	0.21	0.28	0.41
	Thermoleophilia								
		Gaiellales	0.02	0.07	0.00	0.00	0.13	0.04	0.13
		Solirubrobacterales	0.00	0.12	0.00	0.00	0.11	0.09	0.20
Armatimonadetes									
	Armatimonadia								
		Unclassified	0.02	0.09	0.00	0.00	0.06	0.42	0.11
BD1-5									
	Unclassified		0.47	0.38	0.25	0.00	0.00	0.00	0.00
Bacteroidetes									
	Bacteroidia								
		Bacteroidales	2.04	1.55	0.67	0.00	0.00	0.00	0.04
	Cytophagia								
		Cytophagales	0.11	0.28	0.21	0.00	0.32	0.05	0.19
		Order III	0.05	0.17	0.25	0.00	0.00	0.00	0.00
	Flavobacteriia								
		Flavobacteriales	0.18	0.74	0.62	0.00	0.49	0.63	0.74
	Sphingobacteriia								
		Sphingobacteriales							
			0.22	0.88	0.46	0.00	1.90	0.89	2.04
ODI									
	Unclassified		0.20	0.02	0.48	0.00	0.00	0.00	0.00
Chlamydiae									
	Chlamydiae								
		Chlamydiales	0.27	0.17	0.05	0.00	0.13	0.05	0.23
Chlorobi									
	Chlorobia								
		Chlorobiales	0.00	0.00	0.00	0.00	0.00	0.26	0.03
	Ignavibacteria								
		Ignavibacteriales	0.11	0.10	0.14	0.00	0.00	0.00	0.01
Chloroflexi									
	Anaerolineae								
		Anaerolineales	0.09	0.31	0.12	0.00	0.67	1.09	1.17
	Chloroflexia								
		Chloroflexales	0.00	0.00	0.00	0.00	0.08	0.00	0.15
	Gitt-GS-136								
		Unclassfied	0.51	2.31	2.70	0.00	0.74	0.32	1.03

	JG30-KF-CM66								
		Unclassified	1.23	1.40	2.21	0.00	0.19	0.00	0.43
	S085								
		Unclassified	0.72	0.88	0.00	0.05	0.25	0.05	0.43
	SAR202`								
		Unclassified	7.49	5.88	7.03	0.00	0.30	0.44	0.38
	TK10								
		Unclassified	0.04	0.36	0.18	0.00	0.21	0.95	0.73
			0.23	0.26	2.07	0.00	0.00	0.00	0.00
			0.20	0.02	0.16	0.00	0.00	0.00	0.00
	Unclassified								
			0.43	0.28	2.24	0.00	0.00	0.00	0.00
Cyanobacteria									
	Melainabacteria								
		Caenarcaniphilales	0.05	0.19	0.07	0.00	0.02	0.00	0.07
Deferribacteres									
	Deferribacteres								
		Deferribacterales	0.05	0.03	0.14	0.00	0.00	0.00	0.00
Deinococcus-Thermus									
	Deinococci								
		Deinococcales	0.00	0.00	0.05	0.00	0.42	0.11	0.43
Elusimicrobia									
	Elusimicrobia								
		FCPU453	0.00	0.05	0.25	0.00	0.00	0.00	0.00
		Lineage IIc	0.14	0.28	0.09	0.00	0.00	0.00	0.00
		Lineage IV	0.07	0.02	0.65	0.00	0.00	0.00	0.00
		MVP-88	0.02	0.17	0.00	0.00	0.00	0.00	0.00
Firmicutes									
	Bacilli								
		Bacillales	3.57	5.02	4.45	0.70	0.72	0.00	0.88
		Lactobacillales	6.01	6.18	6.66	37.26	0.34	0.28	0.37
	Clostridia								
		Clostridiales	0.14	0.05	0.02	0.00	0.10	0.04	0.10
	Erysipelotrichia								
		Erysipelotrichales	0.00	0.00	0.12	0.00	0.00	0.00	0.00
	Negativicutes								
		Selenomonadales	3.82	4.73	1.64	0.00	0.00	0.00	0.00
Fusobacteri									
	Fusobacteriia								
		Fusobacteriales	3.54	6.40	6.98	0.00	0.00	0.00	0.00
Gemmatimonadetes									
	Gemmatimonadetes								
		AT425-EubC11 terrestrial group	0.00	0.00	0.02	0.00	0.02	0.00	0.13
		Gemmatimonadales	0.13	0.21	0.35	0.00	0.61	0.30	0.54

		S0134 terrestrial group	0.00	0.24	0.12	0.00	0.08	0.00	0.09
JL-ETNP-Z39									
	Unclassified		0.00	0.00	0.00	0.00	0.04	0.00	0.12
NPL-UPA2									
	Unclassified		0.51	0.74	1.29	0.00	0.29	1.30	0.67
Nitrospirae									
	Nitrospira								
		Nitrospirales	1.86	2.48	1.41	0.00	0.80	0.26	0.73
Omnitrophica									
	Unclassified		5.63	5.92	8.41	0.00	0.74	1.16	1.32
Planctomycetes									
	BD7-11								
		Unclassified	0.04	0.21	0.00	0.00	0.00	0.00	0.13
	OM190								
		Unclassified	0.60	0.74	0.00	0.00	0.36	0.00	0.23
	Phycisphaerae								
		CCM11a	0.16	0.26	0.48	0.00	0.00	0.18	0.02
		Phycisphaerales	0.00	0.12	0.00	0.00	0.17	0.67	0.30
		WD2101 soil group	0.00	0.00	0.00	0.00	0.30	0.00	0.48
		mle1-8	0.00	0.00	0.00	0.00	0.00	0.00	0.02
	Pla4 lineage								
		Unclassified	1.48	1.26	2.24	0.00	0.02	0.00	0.12
	Planctomycetacia								
		Planctomycetales	0.63	1.52	0.53	0.00	2.34	4.23	4.76
Proteobacteria									
	Alphaproteobacteria								
		Caulobacterales	0.05	0.03	0.07	0.00	0.40	0.37	0.37
		Rhizobiales	0.31	0.17	0.30	0.25	0.13	0.28	0.36
		Rhodospirillales	0.18	0.09	0.02	0.00	0.06	0.09	0.15
		Rickettsiales	0.25	0.03	0.00	0.00	0.02	0.07	0.00
		Sphingomonadales	0.49	0.09	0.00	0.00	1.29	1.67	1.51
	Betaproteobacteria								
		Burkholderiales	0.22	0.43	0.00	0.08	4.85	4.08	3.59
		Neisseriales	6.01	7.59	2.72	6.23	0.30	0.00	0.07
		Nitrosomonadales	0.40	0.36	0.25	0.00	0.10	0.00	0.37
		Rhodocyclales	0.00	0.00	0.00	0.00	0.11	0.49	0.66
		TRA3-20	0.00	0.16	0.09	0.00	0.00	0.00	0.02
	Deltaproteobacteria								
		Bdellovibrionales	0.20	0.33	0.21	0.00	0.04	0.00	0.12
		Desulfobacterales	0.16	0.09	0.09	0.00	0.06	0.09	0.07
		Desulfurellales	0.81	0.69	0.44	0.00	0.29	0.00	0.38
		Desulfuromonadales	0.40	0.10	0.85	0.00	0.00	0.00	0.01
		GR-WP33-30	0.22	0.24	0.37	0.00	0.08	0.00	0.30
		Myxococcales	4.27	3.81	2.90	0.00	2.76	2.88	2.34

	Epsilonproteobacteria								
		Campylobacterales	0.27	0.10	0.00	0.00	0.00	0.00	0.00
	Gammaproteobacteria								
		Acidithiobacillales	0.01	0.02	0.00	0.00	**61.68**	**58.73**	**48.26**
		Alteromonadales	0.49	0.98	1.01	5.37	0.11	0.09	0.20
		Chromatiales	1.52	2.09	3.64	0.00	0.00	0.00	0.11
		Enterobacteriales	0.00	0.00	0.48	2.26	0.02	0.00	0.00
		KI89A clade	0.29	0.00	0.00	0.00	0.00	0.00	0.00
		Legionellales	0.67	0.45	0.83	35.50	0.13	1.17	0.45
		NKB5	0.05	0.02	0.00	0.00	0.10	0.00	0.01
		Oceanospirillales	0.00	0.00	0.18	7.03	0.06	0.00	0.10
		PYR10d3	0.00	0.00	0.00	0.00	0.30	0.00	0.01
		Pasteurellales	0.52	0.41	0.71	0.00	0.00	0.00	0.00
		Pseudomonadales	18.36	4.16	4.75	0.00	0.49	0.81	1.16
		Thiotrichales	0.00	0.00	0.00	0.00	0.46	0.61	0.00
		Vibrionales	0.00	0.00	0.00	2.74	0.25	0.00	0.00
		Xanthomonadales	4.46	3.16	4.68	0.00	3.22	4.07	3.29
		Unclassified	0.02	0.00	0.21	0.00	0.25	0.05	0.15
SHA-109									
	Unclassified		0.00	0.00	0.00	0.00	0.15	1.81	0.35
Thermotogae									
	Thermotogae								
		Thermotogales	0.14	0.17	0.12	0.00	0.00	0.00	0.02
Verrucomicrobia									
	Opitutae								
		Opitutales	0.09	0.14	0.00	0.00	0.10	0.00	0.47
	Spartobacteria								
		Chthoniobacterales	0.04	0.00	0.00	0.00	0.30	0.00	0.72
	Verrucomicrobia Incertae Sedis								
		Unclassified	0.52	0.66	0.00	0.00	0.08	0.00	0.18
			0.00	0.00	0.02	0.00	3.71	2.77	6.81
Unassigned			4.04	4.55	7.26	0.00	0.70	0.58	1.51

* Data only shown for representative OTUs above the 0.1% threshold
